# Recessive multiple epiphyseal dysplasia (rMED) with homozygosity for C653S mutation in the *DTDST *gene - Phenotype, molecular diagnosis and surgical treatment of habitual dislocation of multilayered patella: Case report

**DOI:** 10.1186/1471-2474-11-110

**Published:** 2010-06-03

**Authors:** Timo Hinrichs, Andrea Superti-Furga, Wolf-Dieter Scheiderer, Luisa Bonafé, Rolf E Brenner, Thomas Mattes

**Affiliations:** 1 Department of Sports Medicine and Sports Nutrition, University of Bochum, 44780 Bochum, Germany; 2 Center for Pediatrics and Adolescent Medicine, University of Freiburg, 79095 Freiburg, Germany; 3 Rehabilitation Hospital Saulgau, 88348 Bad Saulgau, Germany; 4 Division of Molecular Pediatrics, Centre Hospitalier Universitaire Vaudois, 1011 Lausanne, Switzerland; 5 Department of Orthopaedics, Division for Biochemistry of Joint and Connective Tissue Diseases, University of Ulm, 89081 Ulm, Germany; 6 Department of Orthopaedics, University of Ulm, 89081 Ulm, Germany

## Abstract

**Background:**

Multiple epiphyseal dysplasia (MED) is one of the more common generalised skeletal dysplasias. Due to its clinical heterogeneity diagnosis may be difficult. Mutations of at least six separate genes can cause MED. Joint deformities, joint pain and gait disorders are common symptoms.

**Case Presentation:**

We report on a 27-year-old male patient suffering from clinical symptoms of autosomal recessive MED with habitual dislocation of a multilayered patella on both sides, on the surgical treatment and on short-term clinical outcome. Clinical findings were: bilateral hip and knee pain, instability of femorotibial and patellofemoral joints with habitual patella dislocation on both sides, contractures of hip, elbow and second metacarpophalangeal joints. Main radiographic findings were: bilateral dislocated multilayered patella, dysplastic medial tibial plateaus, deformity of both femoral heads and osteoarthritis of the hip joints, and deformity of both radial heads. In the molecular genetic analysis, the *DTDST *mutation g.1984T > A (p.C653S) was found at the homozygote state. Carrier status was confirmed in the DNA of the patient's parents. The mutation could be considered to be the reason for the patient's disease. Surgical treatment of habitual patella dislocation with medialisation of the tibial tuberosity led to an excellent clinical outcome.

**Conclusions:**

The knowledge of different phenotypes of skeletal dysplasias helps to select genes for genetic analysis. Compared to other *DTDST *mutations, this is a rather mild phenotype. Molecular diagnosis is important for genetic counselling and for an accurate prognosis. Even in case of a multilayered patella in MED, habitual patella dislocation could be managed successfully by medialisation of the tibial tuberosity.

## Background

Multiple epiphyseal dysplasia (MED) is a generalised skeletal dysplasia that is clinically and genetically heterogenous. Due to its clinical heterogeneity, it can be difficult to diagnose [1]. Mutations of at least six separate genes can cause the disease: cartilage oligomeric matrix protein (*COMP*) gene [2,3], type IX collagen genes (*COL9A1*, *COL9A2*, *COL9A3*) [4-6], matrilin 3 gene (*MATN3*) [7,8], and diastrophic dysplasia sulfate transporter gene (*DTDST*) [9-11]. Mutations in the first five genes result in autosomal dominant MED, mutations in the *DTDST *gene cause autosomal recessive MED (rMED). DTDST is a sulfate transporter required for the synthesis of sulfated proteoglycans in the cartilage [12]. Over 30 different mutations of the *DTDST *gene have been described [13]. Ballhausen et al. [10] reported on a group of 18 subjects with a recessively inherited form of MED who were homozygous for the mutation g.862C > T (p.R279W) in the *DTDST *gene. Due to this large number of subjects with the same causative mutation, a comprehensive assessment of the particular rMED phenotype was possible. So far, only three cases of rMED with homozygosity for the mutation g.1984T > A (p.C653S) in the *DTDST *gene have been described in the international literature [9]. Here we report on another case of this specific type of rMED.

Clinical characteristics of MED have a wide variation. Very mild phenotypes can even escape medical attention. Short stature, joint deformities, joint pain, gait disorders, irregular epiphyses of tubular bones, and early-onset osteoarthritis are common symptoms. The multilayered patella, first described by Büttner [14] is a rare disorder of the patella, mostly associated with recessive MED. Mutations of *COMP *and *COL9A2 *have been reported as additional causes of multilayered patella [15,16]. Typically the patella consists of two or more layers separated by soft tissue. Clinical symptoms range from asymptomatic forms to chronic dislocation with functional impairment and anterior knee pain. We report on a patient suffering from clinical symptoms of rMED with habitual dislocation of a multilayered patella on both sides, on the surgical treatment and on short-time clinical outcome.

## Case Presentation

### Patients' History

The 27-year-old male patient was admitted to the orthopaedic university clinic because of suffering from pain, swelling and impairment in daily life activities caused by habitual dislocation of the patella on both sides since childhood.

At the age of five years on the left knee an Ali Krogius [17] procedure had been performed without permanent success. Briefly, this is a plastic operation where a strip of medial capsule with the attachment of the vastus medialis muscle is developed. The myofascial strip is then moved over the patella and sutured into a lateral defect that has been created by lateral longitudinal release and medial plication.

The patient reported the "giving way", "jumping" and "snapping" of both knees. He could easily provoke dislocation of the kneecaps and relocate them by himself with only little pain. Furthermore the patient also reported suffering from pain around both hips during activity and during rest. Activities of daily living were only marginally restricted by a flexion contracture of both elbow joints and an extension contracture of both second metacarpophalangeal joints (MCP II). The patient sometimes suffered from mild low back pain. Conservative treatment with physical therapy and muscle strengthening had been effective for pain in both hips but not for patella dislocation.

The patient was of Caucasian origin. He was childless. He had one brother (180 cm/78 kg) who had a sternal protrusion (pectus carinatum). No further skeletal abnormalities or joint symptoms were known in the family. The mother was 162 cm (56 kg), the father 175 cm (87 kg) tall. The patient had a university degree and was working as an engineer. The diagnosis of a "skeletal dysplasia", without further specification, had been made in early childhood.

### Physical Examination

The patient was 170 cm tall, which is between the third and tenth centile of 18-year-old German boys [18]. There was no skeletal disproportion. His body weight was 59 kg. The external ears were normal. The patient had a mild lumbar scoliosis and a pectus carinatum. There was an extension deficit of both elbow joints (30° on the right side, 35° on the left side). Pronation was limited to 60°; supination was limited to 70° bilaterally. There was a flexion deficit of MCP II on both sides. Otherwise, hands were normal. Range of motion of the hip joints was reduced to: extension/flexion 0-0-115° on the right side, 0-0-110° on the left side, internal rotation/external rotation 20-0-30° on the right side, 15-0-30° on the left side, abduction/adduction 30-0-15° bilaterally. Hip rotation and abduction were painful bilaterally. Thigh muscles were atrophic on both sides. Extension/flexion in both knees was 0-0-130°. Both knees had slightly valgus angulations. There was no effusion, no hyperthermia, but slight synovial swelling detectable on either side. Both femorotibial joints were medially, laterally and anteriorly instable. Both patellofemoral joints were instable so that the patient was able to perform an intentional and nearly painless lateral dislocation and relocation of both patellae. The patient had a pes valgus and planus on both sides. The third toe was shortened in comparison to the other toes bilaterally.

### Radiographic Findings

**Figure 1:**
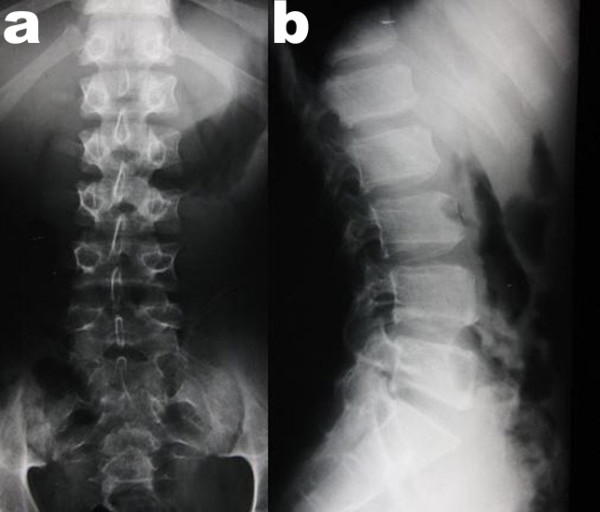
**X-rays of the lumbar spine (a: anterior posterior and b: lateral view) demonstrating mild lumbar scoliosis and defects of the anterior cortical rims of the vertebral endplates**.

**Figure 2:**
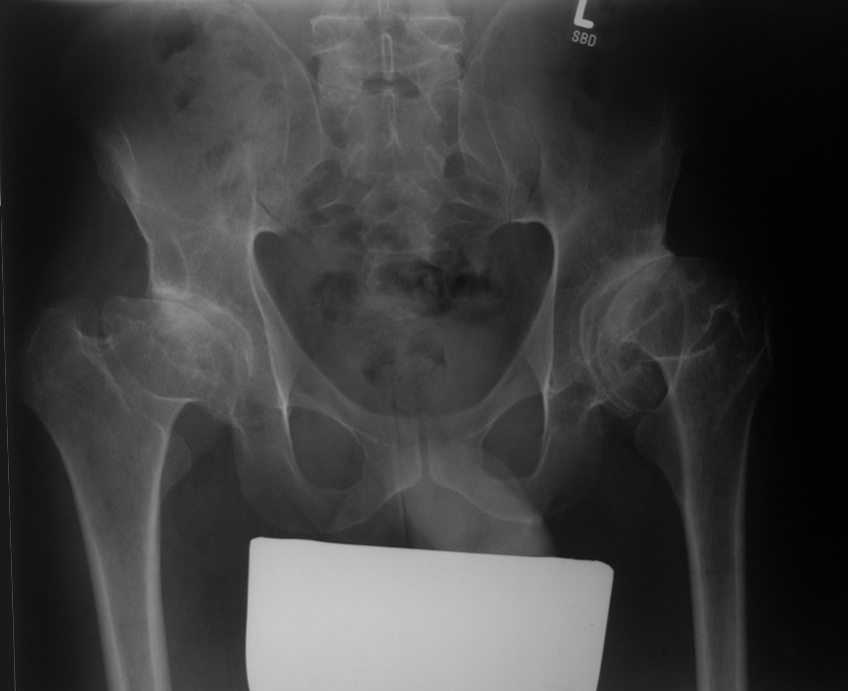
**X-ray of the pelvis (anterior posterior view) demonstrating deformity of the femoral heads and osteoarthritis of the hip joints**.

**Figure 3:**
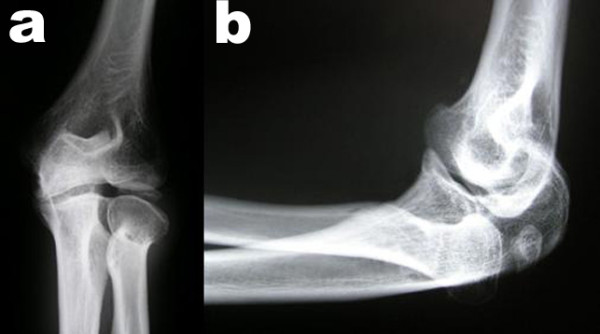
**X-rays of the left elbow (a: anterior posterior and b: lateral view) demonstrating dysplastic deformity of the radial head**.

**Figure 4:**
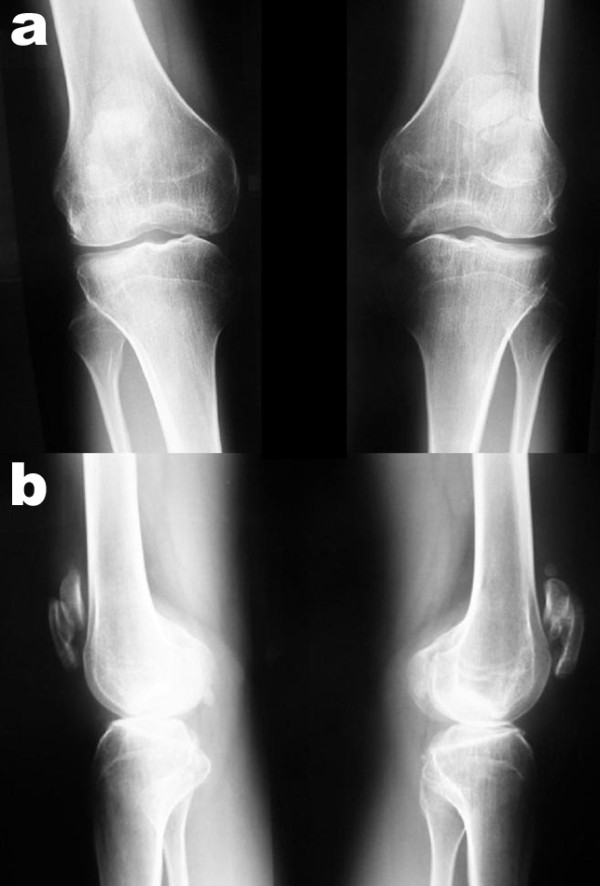
**Preoperative x-rays (a: anterior posterior and b: lateral views) of both knees demonstrating multilayered patellae and dysplastic medial tibial plateaus**.

Radiological examinations of the spine and the pelvis, elbows, hands, and knees were performed. Mild lumbar scoliosis was observed (Figure 1a). Structural changes of the vertebral bodies were limited to defects of the anterior cortical rims of the vertebral endplates (Figure 1b). Radiological examination of the pelvis showed deformity of the femoral heads, shortened and broadened femoral necks and bilateral osteoarthritis of the hip joints (Figure 2). Radiological examinations of the elbow joints revealed dysplastic deformity of the radial head on both sides (Figure 3a, b). The hands showed narrowed joint spaces of MCP II bilaterally, but no further deformity. Radiological examinations of the knees revealed multisegmental patellae, consisting of anterior and posterior components divided by a coronal septum (Figure 4a, b). This finding has been described before as "double-layered" or "multilayered" patella [16,19]. Both medial tibial plateaus were dysplastic (Figure 4a, b).

### Molecular Genetic Analysis

A genomic DNA sample was analysed for *DTDST *mutation because of clinical and radiographic signs similar to reported cases of rMED [9,10]. The *SLC26A2 *(*DTDST*) gene was amplified by PCR and screened for mutations by restriction enzyme digestion and gel electrophoresis with positive and negative controls. Subsequently, selective fragments of the gene were analyzed by bidirectional fluorescent direct sequencing. Results were confirmed in a second amplification product. The *DTDST *mutation g.1984T > A (p.C653S) was found at the homozygote state. Carrier state was confirmed in the DNA of the patient's parents and brother. The identical mutation had been described in three cases with rMED before [9] so that this could be considered to be the reason for the patient's disease.

### Treatment of Patella Dislocation

**Figure 5:**
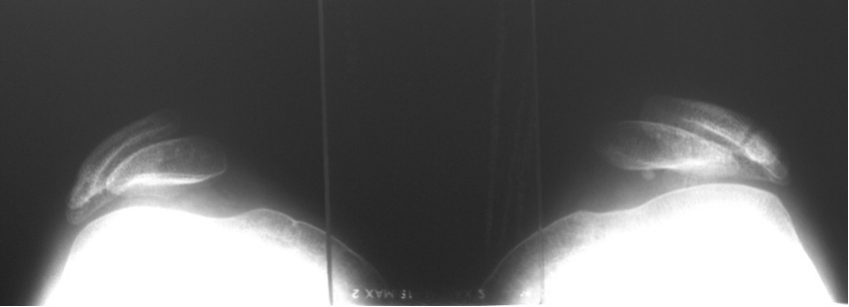
**Preoperative x-ray (skyline view (60° flexion)) of both knees demonstrating subluxation of both patellae**.

Indication for surgery was ongoing recurrent pain and swelling, as well as giving way, with limitation in daily activities without improvement due to conservative treatment. Chronic patella dislocation was treated surgically by medialisation ot the tibial tuberosity in the "Blauth technique" [20] in combination with medial capsular lamination according to Insall et al. [21] starting with the more symptomatic left side. The opposite side was treated one year later. On the left knee with previous surgery a release in the lateral scar was additionally necessary. Patellar cartilage showed minimal degeneration, grade 1-2 according to Outerbridge and Dunlop [22] on both sides. Cartilage in the dysplastic femoral trochlea was macroscopically in normal condition. Medialisation was adjusted intraoperatively until flexion without dislocation was possible to 90°. The osseous chip was shifted about 1 to 1.5 cm medially, placed in a prepared bony groove and fixed with two bi-cortical screws (Figure 5). Flexion at the end of surgery was possible in both knees up to 120°. No additional treatment of the multilayered patella was performed.

Postoperative knee flexion was externally limited to 70° for 6 weeks. A 3-week inpatient rehabilitation program started immediately after surgery. The program consisted of strengthening, endurance and proprioceptive training. The patient was treated with different modalities of physical therapy, physiotherapy and occupational therapy. He received counselling on future physical activity and exercise. At the end of the program, knee and hip pain had been alleviated.

**Figure 6:**
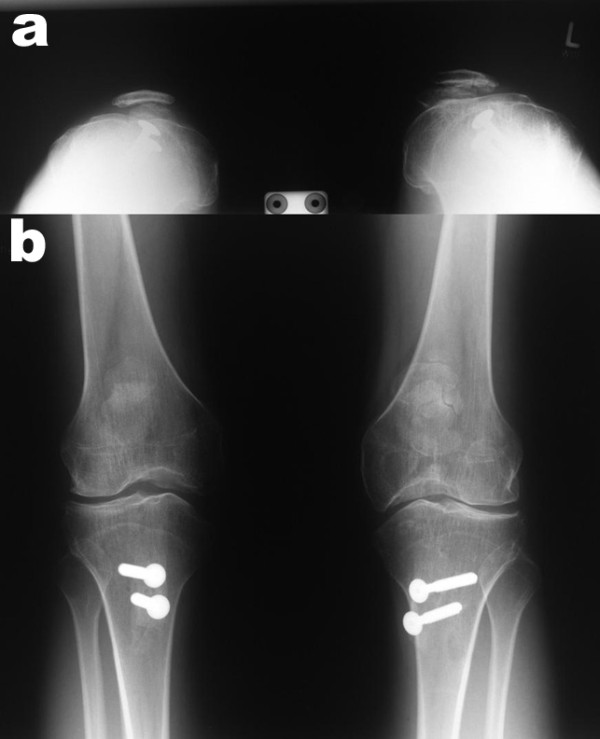
**Postoperative x-ray (a: skyline view (60° flexion) and b: anterior posterior view) of both knees demonstrating well centred patellae**.

One year after surgery on the second (right) knee (two years after the first), the patient was highly satisfied with the results and free from pain in both knees. No re-dislocation of patellae had happened until that time. The clinician rated Knee Society score [23] for the right/left knee was 80/88 (out of 100 possible) points for the "knee score" (including pain, range of motion and stability). The "functional score" (including walking and stair climbing) was 100 (out of 100 possible) points for both knees. Asif and Choon [24] suggested to rate scores higher than 80 points as "excellent" when using the Knee Society score as an outcome measure after total knee replacement. The patient's walking distance was unlimited and he was able to work full time in his job as an engineer. Radiological examination showed centred patellae on both sides and bony fusion of the medialised tuberosity (Figure 6a, b). Screws were removed on both sides simultaneously.

### Discussion of Findings and Therapy

This is the fourth case of rMED with homozygosity for C653S in the *DTDST *gene and the first case of rMED with severe patella dislocation needing medialisation of the tibial tuberosity described in international literature.

The most prominent clinical findings were: bilateral hip and knee pain, bilateral instability of the femoro-tibial and patello-femoral joints with habitual patella dislocation, bilateral contractures of hip and elbow joints, and an extension contracture of both MCP II joints. Main radiographic findings were: bilateral multilayered patellae and dysplastic medial tibial plateaus, dysplastic deformity of both femoral heads and osteoarthritis of the hip joints, deformity of both radial heads, and narrowed joint spaces of both MCP II.

Less prominent findings were a pectus carinatum, a mild lumbar scoliosis, defects of the anterior cortical rims of the vertebral endplates, pes valgus and planus, and shortened third toes. As a pectus carinatum was also known in our patient's brother, who had no further skeletal abnormalities, it was probably not caused by the *DTDST *mutation.

Makitie et al. [9] described three cases of rMED with homozygosity for C653S in the *DTDST *gene before. Like in our case common findings were: bilateral deformation of the femoral heads with secondary osteoarthritis of the hip joints and multilayered, hypermobile patella. In three of four cases there was a bilateral extension contracture of a MCP joint. The following disorders occurred in two cases: flexion contracture in both elbow joints, mild scoliosis, mild dysplastic changes of the vertebral bodies.

Ballhausen et al. [10] reported on 18 cases of rMED with homozygosity for R279W in the *DTDST *gene. The main clinical findings were chronic joint pain, mild brachydactyly, club foot deformity, scoliosis, and joint contractures. The main radiographic findings were flat proximal femoral epiphyses, shortened femoral neck, mild brachydactyly, and multilayered patella. A lateral x-ray of the knee was performed in 10 out of 18 patients; a multilayered patella was observed in seven of them. Ballhausen et al. assumed a high specificity of this sign to recessive MED, as all patients with multilayered patella referred to their institution turned out to have *DTDST *mutations. Nakashima et al. [15] point out that multilayered patella is not exclusive to recessive MED, as they reported a multilayered patella in a male Japanese patient with dominant MED carrying a heterozygous *COL9A2 *mutation. Furthermore, Vatanavicharn et al. [16] described the presence of a multilayered patella also in a patient with pseudoachondroplasia carrying a heterozygous *COMP *mutation.

Summing up, the phenotype of rMED with homozygosity for C653S in the *DTDST *gene seems to resemble that of the patients with R279W rMED. Main common findings are dysplastic hips and multilayered patellae. In contrast to patients with R279W rMED, patients with C653S rMED seem to lack clubfoot deformity and brachydactyly.

Over 30 different mutations of the *DTDST *gene have been described. These result in a continuous clinical spectrum of recessively inherited skeletal dysplasias. The spectrum includes, in order of increasing severity: rMED [9,10], diastrophic dysplasia (DTD) [12], atelosteogenesis type II (AO-II) [25] and achondrogenesis IB (ACG-IB) [26]. While the clinical phenotype in rMED is relatively mild, ACG-IB and AO-II are lethal before or shortly after birth. Patients with DTD usually survive with major physical impairments.

Literature about surgical treatment of double- or multilayered patella is rare. In 1962 Hodkinson [27] described three cases without functional disturbance, without pain and dislocation. A case described by Gasco et al. [28] suffered from giving way and recurrent subluxation. Successful treatment was the excision of the smaller part of the double-layer and a soft tissue procedure described by Insall et al. [21] for stabilisation of the patella. Another case report [29] described anterior knee pain and a painful "snapping" of the patella at 30-40° flexion without dislocation of the patella. A visible jump between the layers was held responsible for the symptoms. The selected surgical solution was a debridement of the soft tissue between the two layers and fusion of the osseous layers with screws. To our knowledge medialisation of the tibial tuberosity has not been described in patella dislocation in MED so far. In our patient soft tissue surgery did not result in stabilisation of the femoro-patellar joint. Therefore, we decided to perform an osseous correction showing good results in non MED populations [20,30-32]. Until now excellent function and painlessness could be achieved. No instability between the layers could be detected clinically in our patient. Thus an additional procedure for bony fusion between the layers was not necessary. Potentially this is only a problem in childhood and adolescents.

## Conclusions

The reported case of rMED demonstrates that homozygosity for C653S mutation in the *DTDST *gene leads to a relatively mild phenotype that seems to be clinically dominated by a tendency to recurrent dislocation of a bilateral multilayered patella and by early-onset osteoarthritis of the hip joints. Conservative treatment of skeletal disorders is in favour for mild symptoms. Nevertheless, surgical therapy of recurrent and severe patella dislocation could be required. Standard techniques, used in patella dislocation in patients without MED, could be performed successfully. In the reported case with severe dislocation a bony procedure with medialisation of the tibial tuberosity was necessary and led to an excellent clinical outcome.

Our patient was diagnosed as "skeletal dysplasia" without any further specification in childhood, but lived his life without any genetic evaluation until he reached the age of 27 years. This underlines the difficulties that medical practitioners might have to diagnose patients with rMED correctly. The knowledge of different phenotypes of skeletal dysplasias helps to determine which genes should be analysed. Molecular diagnosis is important for genetic counselling and for accurate prognosis.

## Competing interests

The authors declare that they have no competing interests.

## Authors' contributions

All authors contributed substantially to acquisition of data: TH, WDS, REB and TM: clinical and radiographic findings; REB, ASF, LB: molecular genetic analysis and interpretation; TM: intra- and peri-operative findings. All authors contributed to conception and design of the manuscript. TH drafted the manuscript. All authors revised the manuscript critically for important intellectual content; and all authors finally approved the version to be published.

## Pre-publication history

The pre-publication history for this paper can be accessed here:

http://www.biomedcentral.com/1471-2474/11/110/prepub
